# RING NMR dynamics: software for analysis of multiple NMR relaxation experiments

**DOI:** 10.1007/s10858-020-00350-w

**Published:** 2020-10-24

**Authors:** Martha A. Beckwith, Teddy Erazo-Colon, Bruce A. Johnson

**Affiliations:** Structural Biology Initiative, CUNY Advanced Science Research Center, 85 St. Nicholas Terrace, New York, NY 10031 USA

**Keywords:** NMR relaxation, Software, Macromolecular dynamics

## Abstract

Molecular motions are fundamental to the existence of life, and NMR spectroscopy remains one of the most useful and powerful methods to measure their rates and molecular characteristics. Multiple experimental methods are available for measuring the NMR relaxation properties and these can require different methods for extracting model parameters. We present here a new software application, RING NMR Dynamics, that is designed to support analysis of multiple relaxation types. The initial release of RING NMR Dynamics supports the analysis of exponential decay experiments such as T_1_ and T_2_, as well as CEST and R_2_ and R_1ρ_ relaxation dispersion. The software runs on multiple operating systems in both a command line mode and a user-friendly GUI that allows visualizing and simulating relaxation data. Interaction with another program, NMRFx Analyst, allows drilling down from the derived relaxation parameters to the raw spectral data.

## Introduction

Molecular motions are fundamental to the existence of life, and NMR spectroscopy remains one of the most useful and powerful methods to measure their rates and molecular characteristics (Palmer 2009; Kleckner and Foster 2011). Two features of NMR are responsible for this value. First, the NMR properties of individual atoms, rather than aggregate properties of an entire molecule, can be measured. These atoms can be of multiple types including commonly used, and structurally important, atoms like carbon, nitrogen, and hydrogen, or atoms used as probes such as deuterium or fluorine. Second, the measurements can be done over a vast range of time scales, from nanoseconds to seconds. These two important attributes mean that NMR can probe dynamics with atomic spatial resolution and with a temporal resolution matched to the biological process in question. This ability to examine different atomic types and different timescales has led to the development of a wide range of NMR experiments, each optimized for a different dynamics phenomenon and different time scale (Sekhar and Kay 2019; Stetz et al. 2019; Marušič et al. 2019).

An individual NMR property, such as the T_2_ relaxation rate constant, has contributions from multiple time scales, and different NMR pulse-sequences have been developed that are appropriate for these different time ranges (Kleckner and Foster 2011). Even within a single NMR property and time range, multiple types of experiments can be performed (cf. R_2_ and R_1ρ_ relaxation dispersion etc. for chemical exchange measurements in the microsecond range). Each different experiment type may require a different method of analysis based on the fundamental equations describing the physical process as coupled to the evolution of nuclear spin magnetization. The complexity of these data has led to development of a wide variety of different software packages for NMR relaxation analysis by various research groups. Even for a single type of experiment, multiple packages exist. Someone analyzing R_2_ relaxation dispersion data can choose from, for example, NESSY (Bieri and Gooley 2011), GUARD (Kleckner and Foster 2012), GLOVE (Sugase et al. 2013), ShereKhan (Mazur et al. 2013), relax (Morin et al. 2014) ChemEx (Bouvignies 2017), catia (Hansen et al. 2008), cpmg_fit (Korzhnev et al. 2007) and others. Ease of analysis and the ability to compare different relaxation parameters on a single system is improved when the application, as with several of these existing programs, can analyze different relaxation experiments. In this way, a user aiming to collect and analyze different relaxation experiments on their molecular system does not need to learn operation of multiple programs, convert their raw data into a variety of different data formats, and possibly run the software on a different OS than they normally use.

We have aimed to develop a new application that builds on advances made in the many existing programs and is designed from the beginning to support multiple types of relaxation experiments. Our new program is cross-platform, so it is functional on MacOS, Windows and Linux operating systems. It has a high-quality graphical user interface and supports multiple relaxation experiment types with a common GUI and data formats. At present it supports analysis of exponential decay experiments such as T_1_ and T_2_, as well as CEST and R_2_ and R_1ρ_ relaxation dispersion. Additional experiment types such as DEST (Fawzi et al. 2011) and model-free analysis capabilities (Mandel et al. 1995) are in development.

## Methods and implementation

The RING NMR Dynamics software is developed in the Java programming language. Using Java allows the software to run unchanged on standard computer operating systems (Windows, MacOS and Linux). An additional advantage of Java is that we are able to easily take advantage of multi-core computers to accelerate the application by running various calculations, such as Monte Carlo simulations, in parallel. Using the new JavaFX GUI toolkit (Weaver and Vos 2012) provides for a high quality, and cross-platform, graphical user interface. The software incorporates Jython, a Java-based implementation of the Python scripting language, to allow for custom scripting (Juneau et al. 2010). Use of the Apache Commons Math library provides access to a variety of mathematical functions, including a variety of methods of numerical optimization. We also include Smile (Statistical Machine Intelligence and Learning Engine, https://haifengl.github.io), which provides capabilities for adding additional analyses based on machine learning.

### Parameter optimization

The primary information derived from the analysis of relaxation data are the parameters that characterize each model, such as relaxation rates, chemical exchange rates, populations, and chemical shift differences between states. These parameters are obtained by optimizing their values to minimize the difference between values calculated from mathematical models and the measured data values. Typical relaxation models contain the parameters as non-linear terms, which precludes the use of ordinary least squares analysis. Non-linear optimizers require initial values of the parameters to be optimized, and convergence of the optimizer to a global minimum may depend on the quality of these values. We have approached this issue in several ways. Previous programs for NMR relaxation analysis often utilize gradient minimizers using algorithms such as Levenberg–Marquardt though some, such as relax (Morin et al. 2014), include non-gradient optimizers. Gradient-based algorithms often require guesses close to the global minimum. We have chosen to use more recent algorithms that tend to have a wider range of convergence. We have implemented both BOBYQA (Powell 2009) and CMA-ES (Auger and Hansen 2005). The latter method, Covariance Matrix Adaptation-Evolution Strategy, is our primary optimizer. The CMA-ES algorithm samples a population of parameter sets and evolves that population towards values that have better fitness. Both of these optimizers support bounds, allowing us to ensure that the minimization converges with physically meaningful parameters. These two algorithms can be slower than gradient-based methods, but besides their good convergence properties, the lack of a requirement for implementing gradient calculations allows for quick implementations of new models.

While CMA-ES and BOBYQA both have good convergence properties, it is still advantageous to start with reasonable guesses. Typical algorithms for relaxation fitting use a grid or random search with bounds to find starting parameters (see for example, GLOVE (Sugase et al. 2013)). We use two alternatives for directly calculating starting values. First, for some equations it is possible to use various approximate rules to come up with good guesses. This is trivial for exponential decay, and reasonable guesses can be made for R_2_ relaxation dispersion fast-exchange equations as well. For example, the lowest measured R_eff_ is a good estimate of the relaxation rate in the absence of exchange, the difference between this value and the largest measured value is a good estimate of R_ex_, and the k_ex_ can be approximately calculated from the midpoint of the dispersion curve.

### Neural network guessing algorithm

More complex relaxation models are less amenable to using this rule-based approach for obtaining initial model parameters. Instead, we have developed a new protocol for estimating the values of the parameters from the experimental data. This protocol uses a multi-layer neural network (Goodfellow et al. 2016) that is trained using simulated data as input and the parameters used to generate the simulated data as test data for output. Training yields a network that, given a set of input data, can generate output parameters close to those used to generate the input. The trained network can then be presented with an experimental data profile and the output values are then good guesses that can be used as input to the numerical optimizers.

Separate neural networks were trained for the fast and slow R_2_ relaxation dispersion models and CEST. The R_2_ relaxation dispersion model training was done using the B_0_ field and R_2eff_ calculated at 10 different values (10.0, 20.0, 50.0, 100.0, 200.0, 400.0, 600.0, 800.0, 1000.0 and 1100.0) of the CPMG field strength (ν_CPMG_) as input values. The network was trained by optimizing the network to reproduce the parameters (k_ex_, ${R}_{2}^{0}$ and ${\delta }_{ppm}^{min}$ for CPMG Fast, and k_ex_, ${R}_{2}^{0}$, p_a_ and δ_ppm_ for CPMG Slow) used to generate the input values. All input values were scaled to fall in a range of approximately 0.0 to 1.0. The CPMG field values were chosen to provide reasonable coverage of typical experiments. Because actual experiments are not necessarily performed at these values, we used interpolation of the measured R_2eff_ values to estimate the R_2eff_ values that would have been measured at the input values used in the training.

Instead of training the network used for CEST parameter estimation on the actual data points of CEST profiles, we used values derived from these profiles. These values were estimated from an analysis of the peak shapes of the CEST profile and included (for both the on-resonance and off-resonance peaks) the widths of the peaks at 25%, 50% and 75% of the maximum depth, the maximum depth, and the baseline (estimated by averaging values at the edges of the profile). The irradiation time (T_ex_) and B_1_ field strength were also used as inputs. As with R_2_ relaxation dispersion training, the input values were scaled to fall in a range of approximately 0.0 to 1.0. The parameters used to generate the input profiles (and used for validating outputs) included k_ex_, p_b_, R_2A_ and R_2B_.

For both the R_2_ relaxation dispersion and CEST networks, we synthesized 10,000 training examples, 3,333 validation data sets and 500 unique testing examples. Each training set was generated by randomly generating values (within appropriate ranges) of the input parameters. A total of 5 layers were used (including input and output layers). The three hidden layers used a ReLU (rectified linear unit (Goodfellow et al. 2016)) activation function and the output layer used a linear activation function.

The number of neurons in the hidden layers was optimized by training five versions of the network and choosing the model that produced the lowest root-mean-square error on the validation data. For each of the five models trained, the number of neurons in the hidden layers were randomly determined, with the constraint that each layer always contained a number of neurons greater than or equal to the number of neurons in the preceding layer. The model which contained the smallest error calculation was returned. The network topologies (number of layers and neurons), the activation functions used, and the weights and biases used to connect each layer were stored in text files that are delivered with the application and loaded on demand during parameter guessing.

### Error analysis

The output of the numerical optimizers described above gives parameter values that best describe the experimental data, but it is also essential to provide information on the confidence of these parameters given the input data. Since its introduction to NMR relaxation analysis protocols in 1991 (Palmer et al. 1991), a standard procedure to generate this confidence information is to use parametric bootstrapping (Hastie et al. 2009). In this protocol, simulated data sets are produced by evaluating the model with the best-fit parameters and then adding normally distributed random noise to the calculated values. These simulated data sets are then fit in the same manner as the original experimental data. For each simulated data set new values of the best-fit parameters are obtained. The distribution, from the set of fits to the simulated data, of each parameter is then used to estimate a confidence value for the parameters. Strictly speaking, with non-linear parameters, this information should be provided with confidence intervals (e.g. 5–95%), rather than a standard deviation which assumes symmetric distributions. We provide the standard deviation of the distribution of parameters, as this is customary in relaxation data fitting.

The above protocol is a parametric method of bootstrapping and requires that some information about the error distribution of the measured values is available. Typically this is obtained by collecting one or more data sets with the same value of independent variable (relaxation time, CPMG strength etc.) (Palmer et al. 1991). The deviation between the replicate values is used to estimate the error distribution for all the values and this error distribution (typically represented as the standard deviation) is used when generating the simulated data sets.

The RING software also implements non-parametric bootstrapping (Hastie et al. 2009). In this method, simulated data sets are generated by randomly choosing, with replacement, from the measured values. These new data sets therefore consist only of actually measured values, but some measured data values may be missing, and some measured values replicated, in any given data set. This protocol has the advantage that no assumptions are necessary about what the error distribution is, or that the error distribution measured at one or a few independent variable values applies to all values, and no experimental time is used in measuring duplicate data points.

The best fit values of the parameters are generally obtained in typical applications by optimizing the parameter values until the sums of the squared deviations between the calculated and measured values are minimized (least squares fitting). This criterion is a requirement of gradient based minimizers such as Levenberg–Marquardt, but is not necessarily the best choice. In particular, least squares fitting is heavily influenced by outliers, as the square of their large deviation contributes disproportionately to the fit. More robust methods, such as minimizing the sum of the absolute values of the deviations, are possible with the non-gradient based optimizers used here. We provide the option of minimizing either the sum of squares or sum of the absolute values of the deviations. In both cases the deviation can be scaled by an error estimate for that value.

### Graphical user interface

**Fig. 1:**
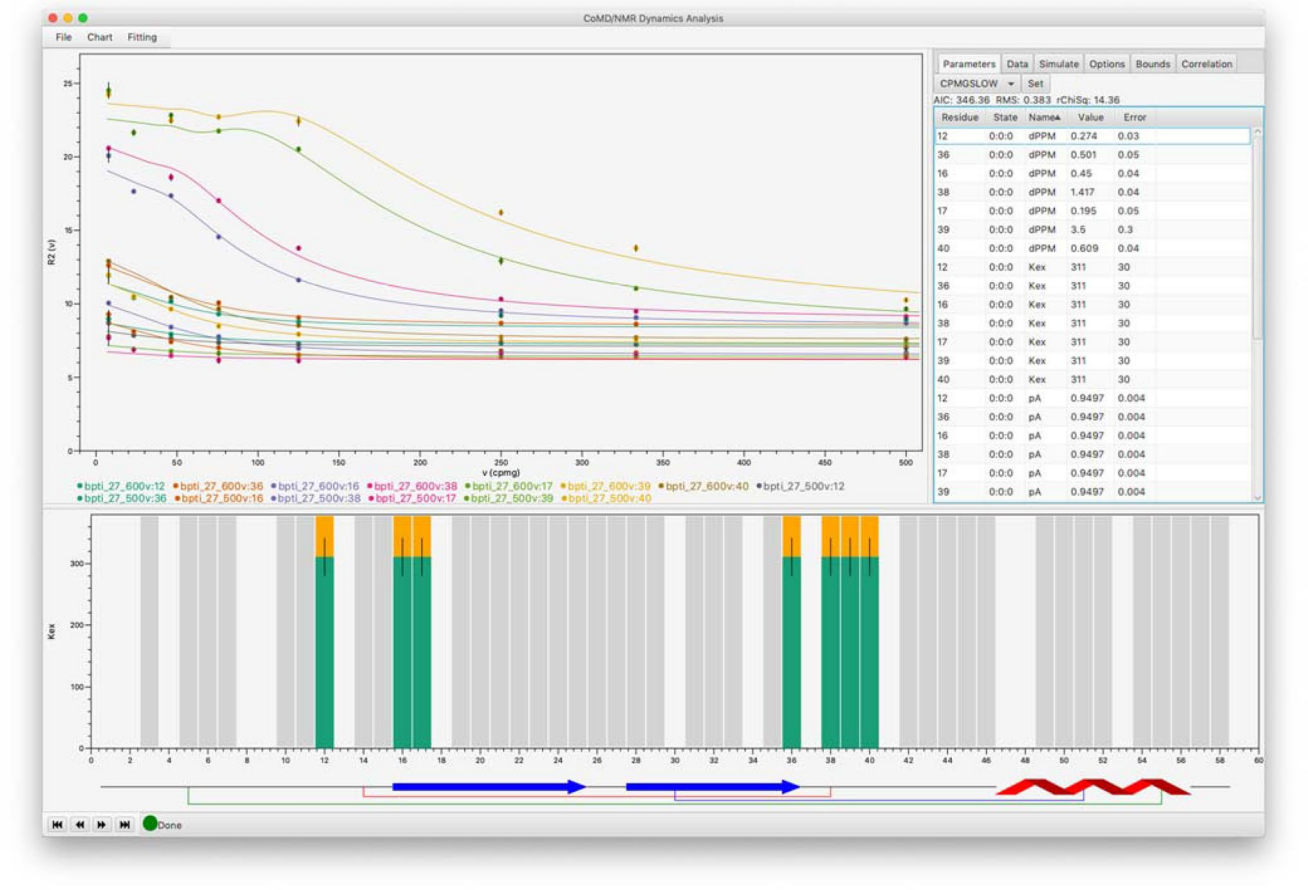
Example of the graphical user interface being used to analyze R_2_ relaxation dispersion data from BPTI. Data values are present for all residues with gray bars, and the data for seven residues are selected for a simultaneous fit. A secondary structure diagram is shown at the bottom, with sheet residues indicated with blue arrows, helical residues with a red helical pattern, and cystine residues in disulfide bonds shown as connected by thin lines

While the software can be run from the command line, and this is useful for batch processing of multiple data sets, a graphical interface provides convenience and data visualization. The GUI is designed to make it easy to set up data analysis and to allow the user to seamlessly see the connections between data values and results. For example, Fig. 1 shows the graphical interface for analysis of R_2_ relaxation dispersion data for BPTI. Clicking on a bar in the residue plot shows the data values for that residue, while clicking on a data point in the XY plot shows the raw data values in the data table. NMR relaxation parameters are often correlated with the secondary structure of the macromolecule, and so we allow display of a graphical representation of protein secondary structures on the residue bar chart.

**Fig. 2:**
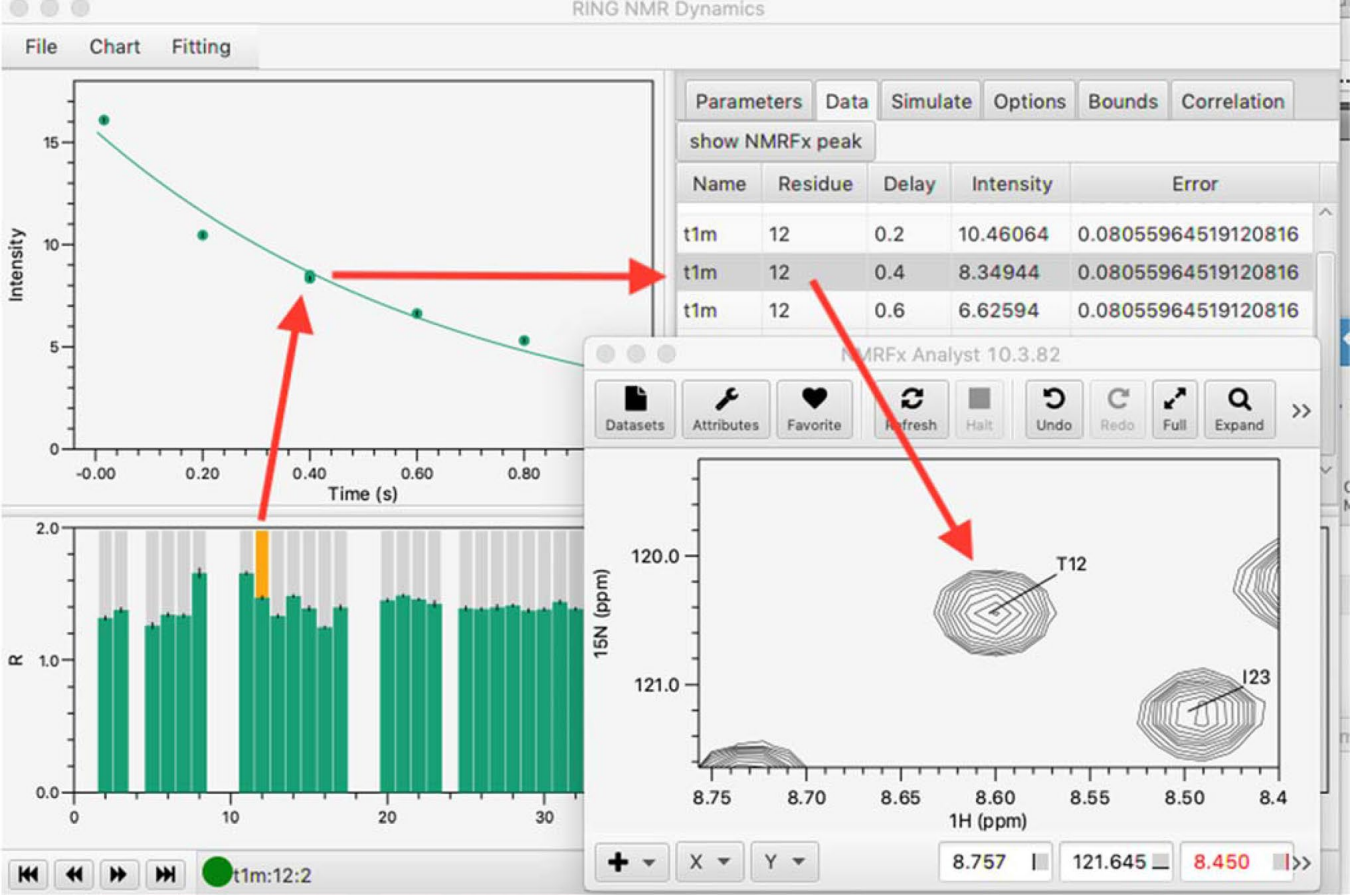
Example of the connection from the RING software to NMRFx Analyst. A residue is selected in the residue bar plot, which results in display of the intensity-time data. Selecting a data point then highlights a row in the Data table. Clicking the “show NMRFx Peak” button drives NMRFx to display the actual spectral data for that peak

The visualization capability has been extended to allow the user to see the raw NMR data in NMRFx Processor (Norris et al. 2016) as well. A listener based on sockets was added to NMRFx, and the RING software can now communicate to this listener through the sockets. A data value in the RING data table, which also includes the NMRFx peak IDs, can be highlighted and the spectral region for the peak is displayed in NMRFx with the click of a button (Fig. 2).

Data can be loaded from several text formats. It is particularly easy to do the peak picking and measurement in NMRFx Processor, as the peak measurement files can be directly read, but data from other programs can be converted into the RING input formats using simple scripts. Sample data sets are also provided for exponential decay, R_2_ relaxation dispersion, CEST, and R_1ρ_ relaxation dispersion profiles.

### Data fitting

Fitting data from individual residues is supported for all experiment types, and global fitting to multiple residues is also possible for R_2_ and R_1ρ_ relaxation dispersion and CEST experiments. In global fitting, parameters such as k_ex_ and excited state populations can be shared by multiple residues.

For R_2_ relaxation dispersion, three models are available to describe the data for the following scenarios: no exchange (${R}_{2}^{eff}={R}_{2}^{0}$), exchange in the fast limit (k_ex_ >  > δω), and when exchange is not in the fast limit. Fast exchange is typically modeled with Eq. (1), where ${R}_{2}^{0}$ is the effective relaxation (${R}_{2}^{eff}$) at infinite CPMG field strength (ν_CPMG_) or where no exchange is present, k_ex_ is the exchange rate, and R_ex_ represents the chemical exchange contribution to transverse relaxation. As seen in Eq. (2) R_ex_ contains contributions from the populations, where p_a_ is the ground or major state and p_b_ is the excited or minor state (p_b_ = 1-p_a_), the chemical shift difference between the two states (δω), and the exchange rate (k_ex_). As the populations and shift difference only appear in Eq. (2) it is not possible in the fast-limit regime to individually extract their contributions. For R_2_ relaxation dispersion data outside the fast exchange limit, data can be fit to a model using the Carver and Richards equation (Carver and Richards 1972) with separate parameters for k_ex_, p_a_, δ_ppm_ and ${R}_{2}^{0}$.

1 $${R}_{2}^{eff}={R}_{2}^{0}+{R}_{ex}\left[1-\frac{4{\nu }_{CPMG}}{{k}_{ex}}tanh\frac{{k}_{ex}}{4{\nu }_{CPMG}}\right]$$

2 $${R}_{ex}=\frac{{p}_{a}{p}_{b}{\delta \omega }^{2}}{{k}_{ex}}$$

3 $${R}_{ex}=\frac{{\left(2\pi {B}_{0}{\delta }_{ppm}^{min}\right)}^{2}}{{4k}_{ex}}$$

4 $${\delta }_{ppm}^{min}=2\sqrt{{p}_{a}{p}_{b}}\left(\frac{\delta \omega }{2\pi {B}_{0}}\right)=2\sqrt{{p}_{a}{p}_{b}}{\delta }_{ppm}$$

A limitation of fitting with R_ex_ as a parameter is that the contribution from the chemical shift difference (in radians/sec) makes it field dependent. The reported value depends on the B_0_ field of the measurement, and when data are acquired at multiple fields R_ex_ is typically reported at one field, making the reported value somewhat arbitrary. Rather than fitting R_ex_ scaled by the field each data set is acquired at, we have chosen to fit to a field independent parameter. In our fitting, the R_ex_ of Eq. (1) is calculated during fitting using Eq. (3), where B_0_ is the field (in MHz) for the measured nucleus and the actual fitted parameter is ${\delta }_{ppm}^{min}$. Substituting the right-hand side of Eq. (3) for R_ex_ in Eq. (2) and rearranging yields Eq. (4). Equation (3) was normalized so that when p_a_ = p_b_ = 0.5 the term to the left of δ_ppm_ in Eq. (4) is 1.0. Thus ${\delta }_{ppm}^{min}$ represents the chemical shift change that would be present if the populations were equal. If the actual populations are unequal the shift difference would be larger, so the value provides the users with an indication of the minimum shift change that must be present (hence the name ${\delta }_{ppm}^{min}$) and is a value that is independent of the measurement field.

During development of the software, we explored and included a wide variety of fitting models for CEST and R_1ρ_ relaxation dispersion. These models are divided into two categories: those that involve an exact solution using integration of the Bloch–McConnell equations, and those that involve various approximations. The models involving approximations run much faster and are useful for exploratory analysis, while the exact solutions are more accurate, but slower. These models are summarized in Table 1. User choices for models can be selected in the user preferences interface and are preserved between sessions.

**Table 1:** Models used for fitting CEST and R_1ρ_ relaxation dispersion data sets

Models	Description	Constraint
Laguerre	Calculates the Laguerre second-order approximation (Miloushev and Palmer 2005) to the eigenvalue and returns R_1ρ_ and then CEST intensity ratio	R_1A_ = R_1B_ R_2A_ = R_2B_
Trott–Palmer	Calculates the perturbation approximation to the eigenvalue (Trott and Palmer 2002; Palmer 2016) and returns R_1ρ_ and then CEST intensity ratio	R_1A_ = R_1B_
Baldwin–Kay	Calculates a first-order approximation to the eigenvalue (Baldwin and Kay 2013) and returns R_1ρ_ and then CEST intensity ratio	R_1A_ = R_1B_
Trott–Palmer-SD	Same as Trott–Palmer above, but varies B_1_ field over a specified range to approximate experimental variance	R_1A_ = R_1B_
EigenExact1	Performs an exact numerical calculation of least negative eigenvalue of Bloch–McConnell rate matrix and returns CEST intensity ratio. Uses matrices and eigenvalue decomposition	R_1A_ = R_1B_
Exact0	Performs an exact numerical integration of thermalized Bloch–McConnell rate matrix and returns CEST intensity ratio. uses matrices and matrix exponential	None
Exact1	Same as Exact0 except for R_1_ constraints	R_1A_ = R_1B_
Exact2	Same as Exact0 except for R_2_ constraints	R_2A_ = R_2B_

This preferences interface also includes options such as the choice of minimizer, bootstrap optimizer, number of bootstrap samples, and fit weighting and tolerances.

When the same data set is fit to multiple models (equations) we provide model selection using the Akaike Information Criterion (AIC) (Cavanaugh and Neath 2019).

### Data visualization

In addition to data fitting, the RING GUI also includes an educational tool to simulate T_1_, T_2_, R_2_ relaxation dispersion, CEST, or R_1ρ_ relaxation dispersion profiles in the absence of experimental data. The shape of the simulated profile can be adjusted using the parameter value sliders, and example data points can also be generated and subsequently fit. This allows the user to get a general idea of how various parameters affect the data profile without having to perform potentially time-consuming fits to actual data.

In order to use the data figures in publications, the RING software allows exporting of charts (both XY and the residue bar chart including the secondary structure) in PNG and SVG (a vector format that preserves full data resolution) formats. The user can also export data into plot files that can be read in the plotting program Grace, and that can be executed in the programming/plotting environments of R and Python. Using the latter two programs allows the user to have programmatic control over all aspects of plotting.

**Fig. 3:**
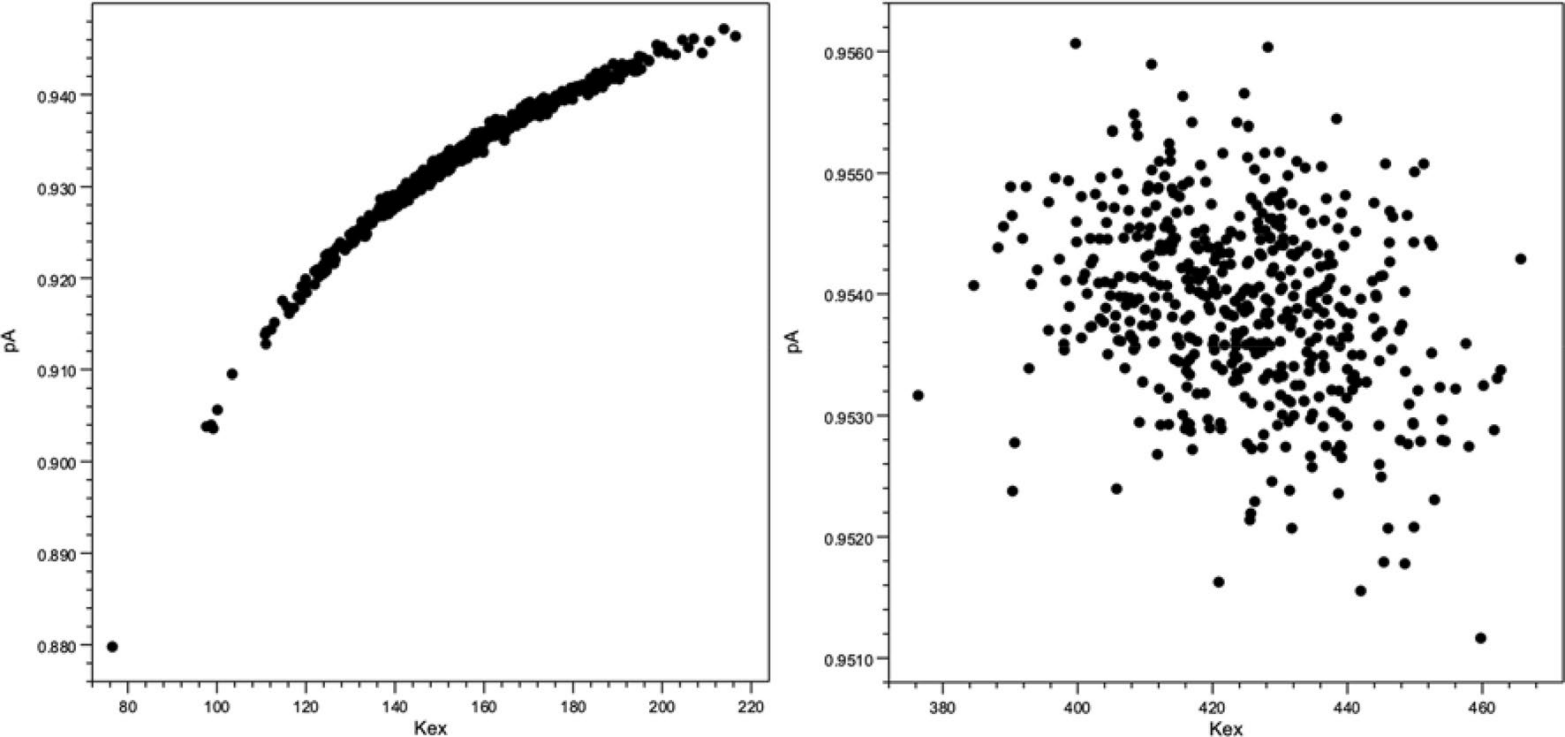
Plots of parameter values generated during the parametric bootstrapping protocol. These plots can be generated interactively within the software after any fitting procedure. The sample data shown here is R_2_ relaxation dispersion data from the sample BPTI data set fit to the slow-exchange model. Data in the left panel was generated from a fit with data at 500 MHz. Data in the right panel is from a simultaneous fit to data at 500 and 600 MHz. The GUI allows the user to plot any pair of parameters. Here the plot of p_A_ and k_ex_ is shown. The data at one field shows that these two parameters are highly correlated, making it difficult to establish a narrow confidence range for either one. Note that the plot limits for these two plots are different. The bootstrapping values at two fields are in a much narrower, and *non-overlapping* range, compared with those values from the single field fit

There can be a high degree of correlation between parameters used in some NMR relaxation models. This is especially true when data are only collected at one field, and it is useful to be able to observe this. One way to visualize parameter correlation is to plot the parameters obtained during the bootstrap procedures described above. The RING software retains all the measured parameters generated in the bootstrapping process and any pair of parameters can be plotted with respect to each other as shown in Fig. 3.

All the RING software is released as open source, using the GNU Public License (GPL v3.0) and is available on GitHub (https://github.com/brucejohnson/ringnmrdynamics). Executables are available at https://comdnmr.nysbc.org/comd-nmr-dissem/comd-nmr-software.

## Results

Figure 1 illustrates the software in action. As can be seen in the figure, the main GUI is divided into three regions. Across the bottom is a bar chart that plots data as a function of residue position. At top left is an XY plot for showing residue specific data. At top right is a tabbed pane with tabs for data display and interactive controls.

After loading in the data, the filled bars (gray fill) in the residue bar chart indicate residues with available data. After performing data fitting to all residues (one option from the Fitting menu), the bars are plotted with values relevant to the data type. For chemical exchange experiments (R_2_ relaxation dispersion, CEST, R_1ρ_ relaxation dispersion) the plot values show the best fit values for the exchange rate. Values are only shown where the AIC model selection protocol has selected a model for the residue with exchange. For T_1_ and T_2_ experiments the plotted value will be the relaxation rate. Bars have a line indicating estimated error for best-fit parameters (where relevant). While the default is to plot k_ex_ or relaxation rates, any residue specific parameters available after fitting can be displayed. These include other parameters from the fitted model as well as values such as the RMS (root mean square deviation of fit) or the AIC. By default, model specific parameters are plotted for the model that was selected (by AIC protocol) for each residue, but the user can choose to plot values for any of the fitted models. Multiple values can be plotted simultaneously either by adding additional rows of bar charts, or by plotting multiple values for each residue.

Macromolecular dynamics are correlated with secondary structure, so it can be useful to show a graphical representation of the secondary structure along with the residue specific values. An example is shown at the bottom of Fig. 1. The plot can represent helices, sheets and lines connecting residues in disulfide bonds. This example shows that the exchanging residues of BPTI are adjacent to a disulfide bond consistent with the dynamics being due to disulfide bond isomerization (Millet et al. 2000).

The data values in the XY plot are typically selected from the interactive simulation controls or, as shown here, by selecting residues in the bar chart. When the user clicks on a bar in the residue plot, the XY data for that residue is displayed. The exact plot depends on the experiment type. Here, the R_2eff_ vs. ν_CPMG_ is plotted. Multiple residues can be selected (seven in this example).

Fitting can be done to all the residues that have data, or to the subset of residues that have been selected for display in the XY chart. When multiple residues are selected the fitting is done simultaneously to the data from the selected residues and appropriate parameters (in Fig. 1, k_ex_ and p_a_) are shared by all selected residues.

The tabbed pane at top right of the GUI has tabs for various data values and controls. After fitting data, the Parameters tab shows a table of all fitted parameters with their best fit values and estimated errors. Using the mouse to click on a data value in the XY chart will update a table in the Data pane with the raw values for that plot.

**Fig. 4:**
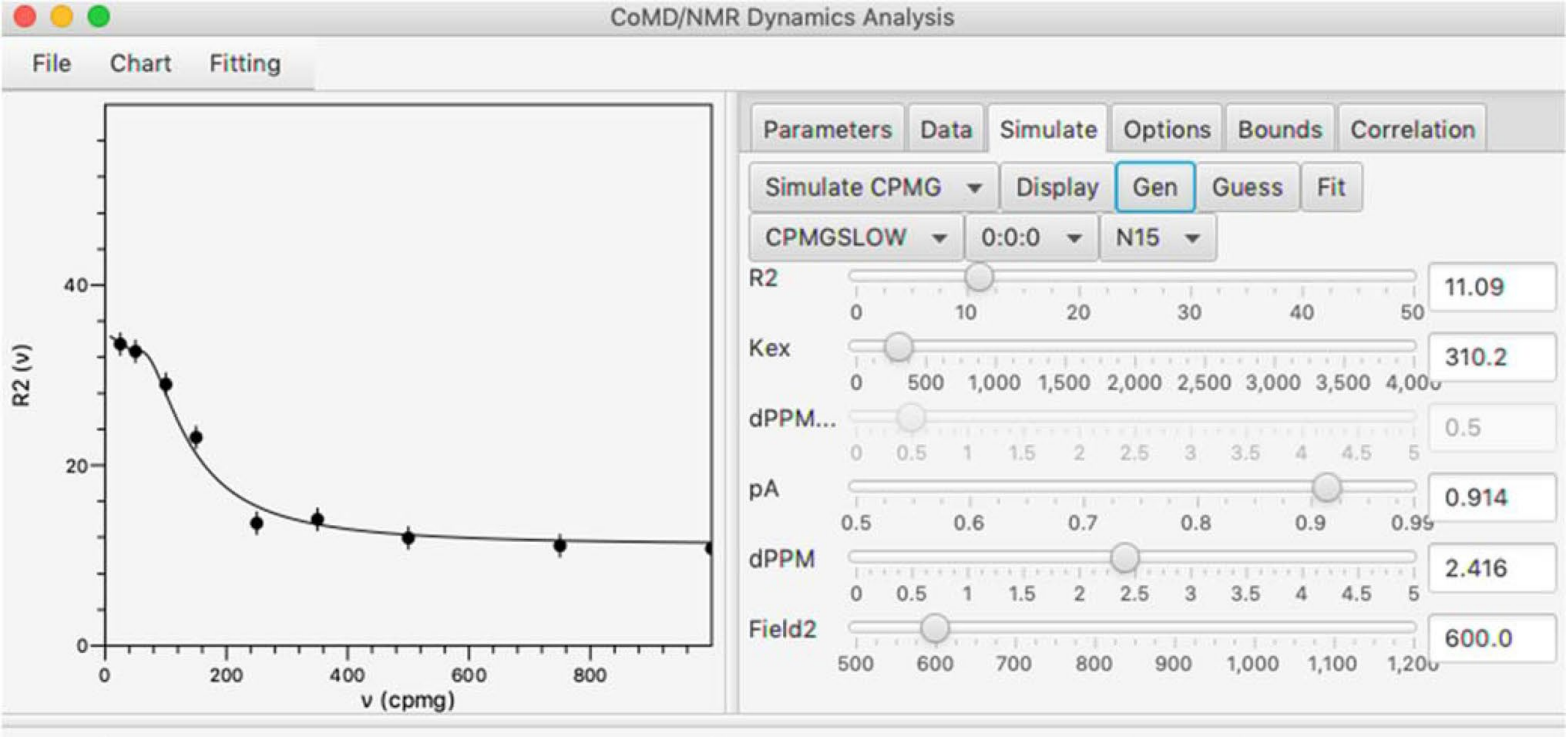
Example of the graphical user interface being used to simulate data. As the sliders in the right side are adjusted, the curve in the plot on the left is updated based on the selected equation and parameter values

A significant goal for development of this application was to make it educational for users new to NMR dynamics. One part of that was to provide the ability to simulate data with interactive controls so that users can observe the effect that changes in parameters have on the profile of experimental data. These controls are available in the Simulate tab and an example of its use is shown in Fig. 4. The user can select the experiment type (R_2_ relaxation dispersion, CEST etc.), the particular model (FAST exchange, SLOW exchange etc.) and nucleus. Appropriate sliders are displayed which can be used to set values for the parameters. As values are adjusted the XY plot will show a line calculated with the current parameters. Simulated experimental data values can be generated (Gen button) with added noise (shown as filled circles in plot). This is useful for testing the guessing (Guess button) and fitting (Fit button) algorithms. Values for the independent variable and amount of added noise can be set in the Options tab.

Relaxation models can be underdetermined by the available data and the model parameters can be highly correlated as a result. Plotting the data from the bootstrapping can be used to visualize this. Figure 3 shows a plot of p_a_ vs. k_ex_ from the 500 Monte Carlo replicates used in the bootstrap estimate of error (Millet et al. 2000; Kovrigin et al. 2006) values from the R_2_ relaxation dispersion data for one residue of BPTI. The left panel (4A) is data from only a single field (500 MHz). In this case the population and exchange rate are highly correlated. The right panel shows the results obtained when data from two fields (500 and 600 MHz) are used. As is well known (Millet et al. 2000; Kovrigin et al. 2006), data from the additional field can significantly constrain the data so, visually, essentially no correlation exists between the values. Importantly, as can be seen from the figure, the fit parameters are essentially non-overlapping between the single and two-field fits. Fitting to two fields constrains the parameters to values that are not included with the single field fit. Thus, this tool provides an important guide to users in evaluating the sufficiency of their data.

**Fig. 5:**
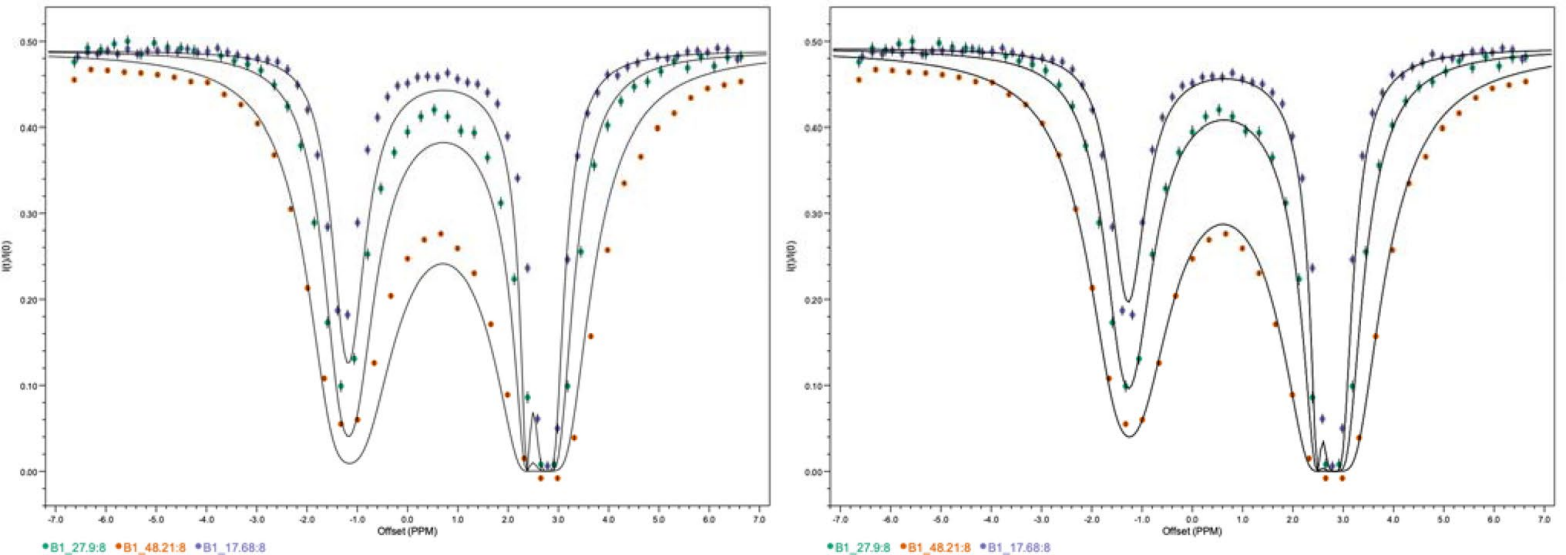
Demonstration of the parameter guessing with the trained neural network. Sample data are CEST profiles from the sample RNA data (Zhao et al. 2014). The solid lines on the left panel are calculated from the parameters output from the neural network with the sample data as input. The solid lines in the right panel represent calculated curves from the best-fit parameters (using the guesses as input)

Reaching the global minimum in fitting non-linear models is dependent on both the optimization algorithm and the quality of the starting guess. We have provided two optimizers, CMA-ES and BOBYQA, that have good convergence properties. We’ve also introduced a new protocol using an artificial neural network (ANN) for generating the initial parameters. Figure 5 shows a CEST data set with the lines showing the model with the initial parameters and final best-fit parameters. As can be seen, the ANN is able to generate guesses that give a good representation of the data without needing to do grid or random searches over parameter space. The network is trained, and predicts, using measurements of the peak widths. These are incomplete representations of the full data, so we do not expect the network to provide best fits by itself.

To validate the software, we have relied on testing against real-world, previously published, data sets. In the sections below, we describe tests using CEST, R_1ρ_ and R_2_ relaxation dispersion data sets.

### CEST performance metrics

Given the wide variety of models available for CEST fitting, we conducted a series of tests to gauge the calculation speed and accuracy for each model. The tests were done using previously published ^13^C CEST data on a fluoride riboswitch (Zhao et al. 2014). All test fits were performed using the CMA-ES optimizer. The speed of the CEST data fitting obviously depends on the equation used. As expected, the equations using approximations were very fast, with best-fit parameters obtained in less than 50 ms. The equations involving exact solutions to the Bloch–McConnell equations were much slower, taking 2–3 s to fit the data. The slower speed is not surprising given the much greater computational burden involved in the exact analysis. The current performance, though slower than the approximations, is significantly better than our original version due to careful optimization of the code.

**Fig. 6:**
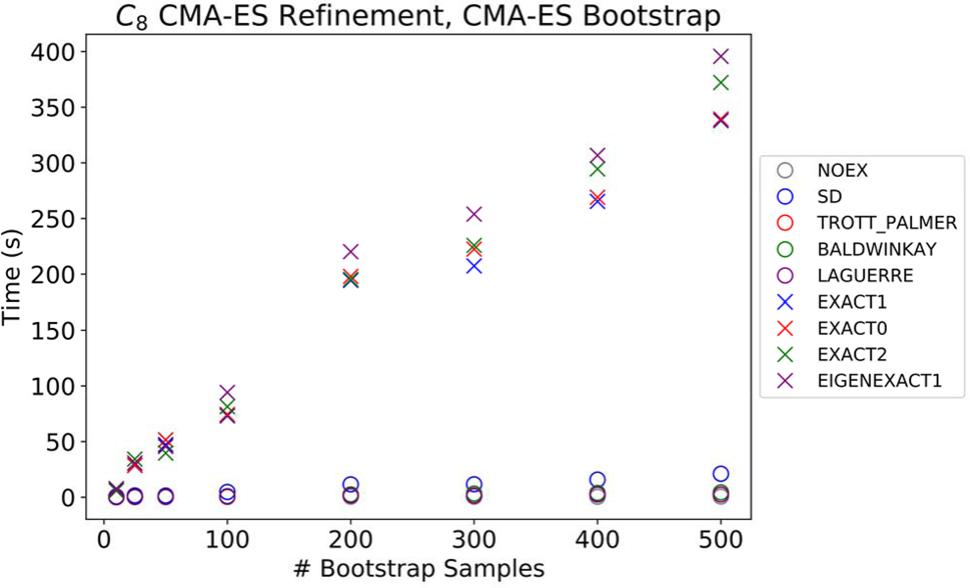
Performance assessment of the CEST data fitting showing the significantly slower fitting obtained with the EXACT equations, and the near-linear increase in time with increasing number of bootstrap samples

To determine how the number of bootstrap samples affects the total time for the error calculation, tests were conducted using a range of 10 to 500 bootstrap samples using the CMA-ES optimizer. The results are plotted in Fig. 6. Regardless of the optimizer or equation used, a near linear relationship is observed between the number of bootstrap samples and the error calculation time: doubling the number of bootstrap samples roughly doubles the time it takes to complete the error calculations. The bootstrap time is, however, significantly faster than expected based on the refinement time. For example, the calculation using 100 bootstrap samples is done in approximately 20 times (rather than 100 times) the time it takes to do one refinement. This is, in part, due to the fact that we use Java threads to run the bootstrapping in parallel, taking advantage of multi-core processors that are typical of modern computers.

Fit accuracy for the CEST equations was also examined with the RNA CEST data (Zhao et al. 2014). To assess the accuracy of the various models we measured the fit quality (rms deviation) and compared the best fit values for all the model parameters to the reported literature values (Table 2). The different models fall into three groups determined by whether approximations are used and what parameters are constrained. The best fits, and closest match to literature data, are obtained with two of the three exact models (EXACT0 and EXACT1). These do a numerical integration of the Bloch–McConnell rate matrix and allow independent values for the ground and excited state R_2_ values. The similar qualities for these two models indicate that constraining R_1_ values to be the same for the ground and excited states (as done in EXACT1) does not reduce the quality of the fit. Intermediate results were obtained with the Trott–Palmer and Baldwin–Kay approximation models, and the EIGEN-EXACT model. The rms deviations are nearly as good as the two exact models, but k_ex_ is larger, and p_b_ a little smaller, than the literature values. The worst fits and largest deviations from literature values are obtained with the Laguerre approximation and EXACT2 models. Both of these models constrain the R_2_ values for the excited and ground states to be the same and this is clearly, unlike constraining R_1_, a hindrance to good quality fits for these data.

**Table 2:** CEST fitting parameters for the sample RNA data

	AIC	RMS	k_ex_	p_b_	δ_A0_	δ_B0_	R_1A_	R_1B_	R_2A_	R_2B_
TROTT–PALMER-SD	− 533	0.014	163.2 ± 24.2	0.08 ± 0.006	2.8 ± 0.02	− 1.3 ± 0.01	2.4 ± 0.01	2.4 ± 0.01	19.4 ± 1.9	148.1 ± 17.5
TROTT–PALMER	− 531	0.014	157.5 ± 20.9	0.08 ± 0.005	2.8 ± 0.02	− 1.3 ± 0.01	2.4 ± 0.01	2.4 ± 0.01	19.2 ± 1.7	155.1 ± 19.2
BALDWIN–KAY	− 528	0.014	164.9 ± 21.4	0.08 ± 0.004	2.8 ± 0.02	− 1.3 ± 0.01	2.4 ± 0.01	2.4 ± 0.01	21.3 ± 1.9	153.0 ± 17.0
LAGUERRE	− 432	0.020	300.9 ± 30.2	0.07 ± 0.005	2.9 ± 0.03	− 1.4 ± 0.03	2.5 ± 0.01	2.5 ± 0.01	11.9 ± 2.2	11.9 ± 2.2
EXACT1	− 648	**0.010**	**109.9 ± 13.6**	**0.10 ± 0.005**	**2.8 ± 0.01**	− **1.3 ± 0.01**	**2.0 ± 0.03**	**2.0 ± 0.03**	**25.6 ± 1.1**	**153.4 ± 11.0**
EXACT0	− 647	0.010	107.5 ± 13.7	0.10 ± 0.006	2.8 ± 0.01	− 1.3 ± 0.01	2.0 ± 0.03	2.2 ± 0.09	25.7 ± 1.2	154.0 ± 9.2
EXACT2	− 518	0.015	243.7 ± 23.5	0.07 ± 0.006	2.8 ± 0.01	− 1.4 ± 0.02	2.2 ± 0.04	2.5 ± 0.37	16.3 ± 1.0	16.3 ± 1.0
EIGENEXACT1	− 519	0.015	161.8 ± 25.2	0.08 ± 0.006	2.8 ± 0.02	− 1.3 ± 0.01	2.4 ± 0.01	2.4 ± 0.01	20.7 ± 2.2	145.6 ± 21.7
Literature			109.0 ± 8.0	0.10 ± 0.003	− 4.09 ± 0.01		2.4 ± 0.01	2.4 ± 0.01	24.1 ± 0.8	147.0 ± 7.0

The best fit to the data is shown in bold. Literature values are for the RNA C_8_ atom at 30 ºC from (Zhao et al. 2014)

Given the better quality fits, but slower performance, of the exact models, a reasonable approach is to use the approximate models like Trott–Palmer or Baldwin–Kay for exploratory analysis (or during use of the simulation tool), and then generate final results with EXACT0 or EXACT1.

Estimation of the uncertainty of the fitted parameters is also important, and we provide two ways of estimating this uncertainty. The values in Table 2 were generated with our non-parametric bootstrapping protocol. It can be seen that the estimated standard deviations are larger than the literature values. For example, the EXACT1 model gives a k_ex_ value of 111 ± 13 whereas the reported value is 112 ± 4. Estimating the errors with parametric bootstrapping using the authors’ error estimates yields values (111 ± 4) consistent with the literature values. The rms deviation of the fit with the EXACT1 model is approximately 0.01. This value is larger than the published error estimates (0.003, 0.006 and 0.007 for the three data series fitted). This suggests that those estimates might underestimate the errors and that the values obtained from non-parametric bootstrapping might be more realistic estimates of the uncertainty in the parameters. In any case, providing both methods of uncertainty estimation is an important feature, and allows for data analysis without explicit measurement of the data errors.

**Fig. 7:**
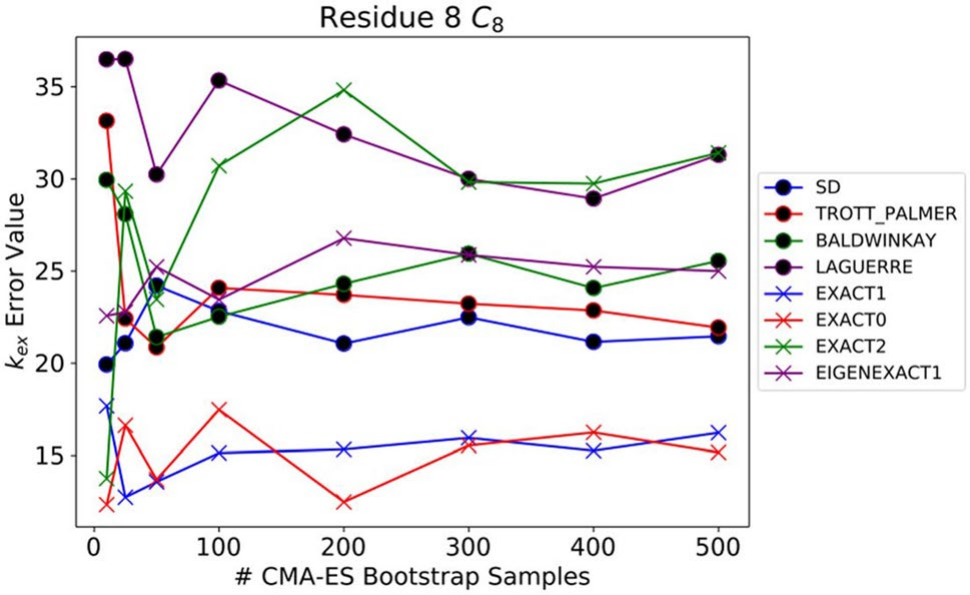
Estimate of the error in the best-fit k_ex_ value for the CEST data as a function of the number of bootstrap samples

While the exact equations yield more accurate values for the CEST parameters, optimization of model parameters is orders of magnitude slower than the fitting using the approximate equations. These differences in timing are particularly pronounced when performing the bootstrap simulations to estimate the uncertainty in the parameters. To determine the number of bootstrap samples needed for accurate parameter error value estimates, we calculated the error values for the RNA CEST fit parameters using bootstrapping with 10, 25, 50, 100, 200, 300, 400, and 500 samples. Figure 7 shows the k_ex_ error values as a function of the number of CMA-ES bootstrap samples for the nine CEST equations. Generally, the error estimates fluctuate up to 50 samples, after which the variability levels off. This suggests that using around 50 samples is sufficient for reasonable error estimates, but the user may wish to run more to get final estimates.

### R_1ρ_ relaxation dispersion performance metrics

The R_1ρ_ relaxation dispersion fitting was tested with the R_1ρ_ data for the same RNA molecule as the CEST data (Zhao et al. 2014). We assessed the accuracy of the various models in the same way as for CEST. Examples of fitted parameters and the reported literature values are shown in Table 3. These data were collected with an experiment in which only ground state magnetization is present at the start of the spin-lock period. As noted by the original authors (Zhao et al. 2014), this leads to an apparent elevation of the baseline relative to what is expected for the R_1_ relaxation rate. Correction for this can be done in two ways. When fitting with the EXACT0 equation, which uses integration of the Bloch–McConnell equations, one needs to ensure that the vector representing starting magnetization is set to be the ground-state population (based on the fitted population parameter). Alternatively, one can calculate a correction factor as previously described (Korzhnev et al. 2005). In that case, the apparent R_1ρ_ is fitted as the sum of the calculated R_1ρ_ and this correction factor. In both these cases it is necessary to constrain the R_1_ relaxation rate to a value obtained by other methods (Zhao et al. 2014). The software described here has a preference value that can be set and controls whether the correction factor is applied.

**Table 3:** R_1ρ_ relaxation dispersion fitting parameters for the sample RNA data

	AIC	RMS	k_ex_	p_b_	δ_A0_	δ_B0_	R_1A_	R_1B_	R_2A_	R_2B_
NOEX	906.4	5.48	0.0 ± 0.0	0.0 ± 0.0	0.2 ± 0.08	0.0 ± 0.0	2.4 ± 0.04	0.0 ± 0.0	43.4 ± 4.6	0.0 ± 0.0
TROTT_PALMER	367.1	0.464	114.3 ± 2.3	0.1 ± 0.002	0.0 ± 0.01	4.0 ± 0.04	2.5 ± 0.03	2.5 ± 0.03	28.7 ± 0.4	81.2 ± 11.4
BALDWINKAY	408.3	0.558	105.2 ± 3.5	0.1 ± 0.002	0.0 ± 0.01	3.9 ± 0.04	2.5 ± 0.02	2.5 ± 0.02	34.2 ± 0.4	101.2 ± 17.2
LAGUERRE	571.2	1.178	117.7 ± 5.1	0.11 ± 0.004	− 0.3 ± 0.02	4.0 ± 0.07	2.5 ± 0.01	2.5 ± 0.01	28.5 ± 1.3	28.5 ± 1.3
EXACT0	**310.8**	**0.36**	**111.6 ± 2.5**	**0.11 ± 0.002**	**0.0 ± 0.0**	**3.9 ± 0.02**	**2.5 ± 0.03**	**2.5 ± 0.03**	**29.0 ± 0.3**	**130.3 ± 20.0**
EIGENEXACT	313.2	0.364	110.7 ± 1.6	0.11 ± 0.002	0.0 ± 0.01	3.9 ± 0.04	2.5 ± 0.03	2.5 ± 0.03	30.3 ± 0.3	67.2 ± 11.5
Literature			116.0 ± 2.0	0.11 ± 0.002	− 3.95 ± 0.02		2.4 ± 0.03	2.4 ± 0.03	28.8 ± 0.2	122.0 ± 15.0

The best fit to the data is shown in bold. Literature values are for the RNA C_8_ atom at 30 °C from (Zhao et al. 2014)

**Fig. 8:**
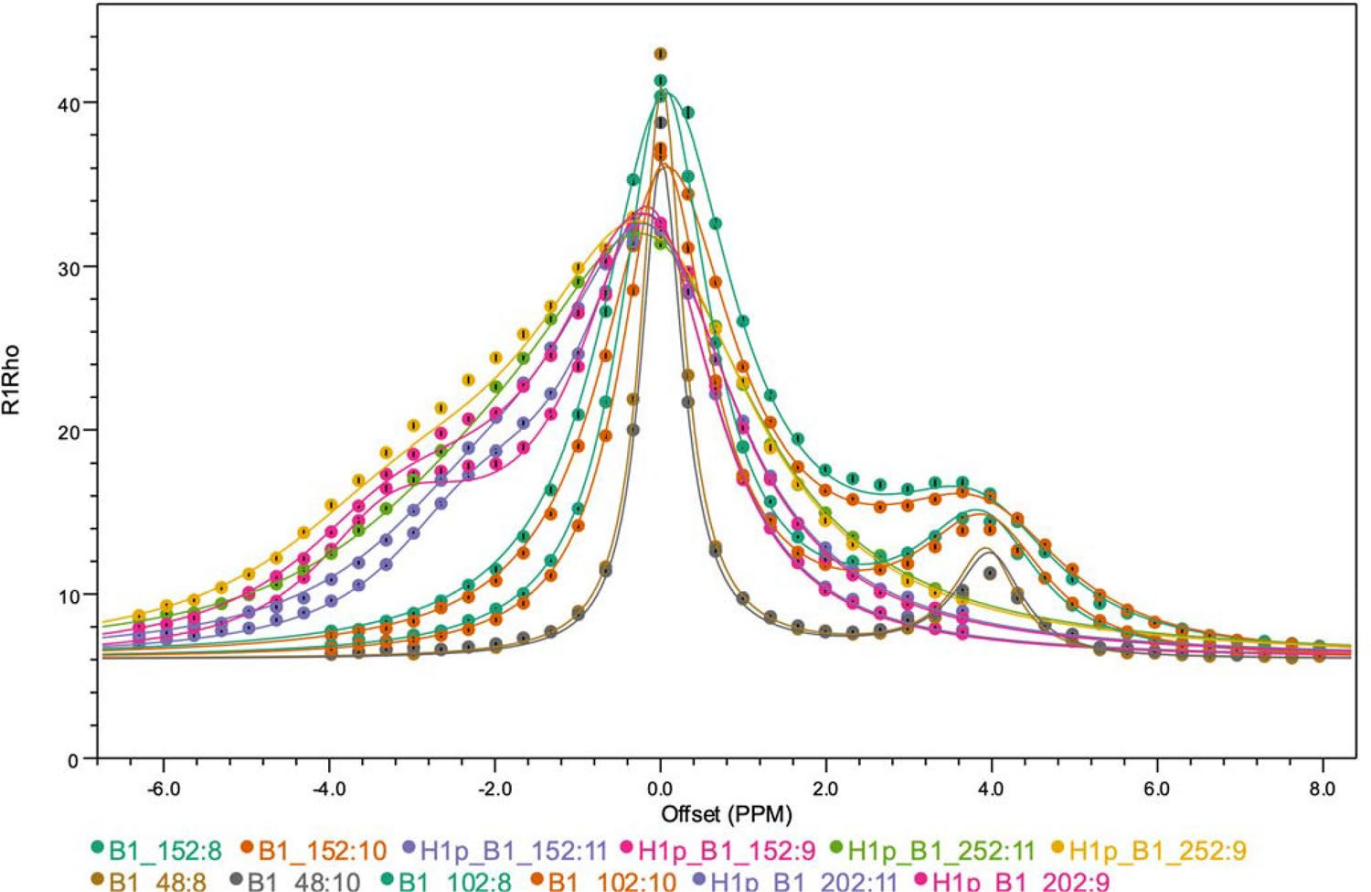
Example of R_1ρ_ profiles fit simultaneously for two atoms from two RNA residues at three different B_1_ field strengths

For this sample R_1ρ_ dataset, the best model, as selected by the lowest AIC value, is the EXACT0 model using integration of the Bloch–McConnell equations. The Trott–Palmer approximate model, however, also has a very good fit and the calculation time is much lower. Both methods give good agreement with the literature values, though R_2B_ is somewhat underestimated with the Trott–Palmer model. As with the CEST and R_2_ relaxation dispersion fitting, it is possible to simultaneously fit multiple data sets. Figure 8 shows the fit to the sample RNA data with measurements from two residues and two atoms on each residue. Values of k_ex_ and p_b_ are globally fit to all data along with 20 other individual parameters describing R_1_, R_2_ and offsets. The resulting parameters have good agreement with the literature values for a global fit.

### R_2_ relaxation dispersion fitting tests

Validation of the R_2_ relaxation dispersion fitting was done by comparison to previously published R_2_ relaxation dispersion data for basic pancreatic trypsin inhibitor (BPTI) (Millet et al. 2000). All residues were fit, but Table 4 shows only results for residue 38 and some global fits of multiple residues. The values shown were chosen to make several points about R_2_ relaxation dispersion fitting. One of these is the ease with which the software can be used to explore fits with different models. The data were fit to both the fast-exchange and slow-exchange models, and at 500 MHz and 600 MHz individually and simultaneously. Comparing the simultaneous 500/600 MHz fits to the two models shows, based on the AIC values, that the slow-exchange model is appropriate for fitting data for this residue. Another point is that commonly used protocols for determining the errors in parameter estimation can be misleading. For example, the k_ex_ values at 500 MHz and 600 MHz differ substantially, both for our fits and the literature values, and the differences far exceed the reported error limits using parametric bootstrapping. The table also shows, for data at 500 MHz, the fit using non-parametric bootstrapping. This fit gives much larger error bars (± 225) than that using the parametric fit (± 11) and is probably a more realistic estimate of the uncertainty in the parameters. The use of parametric fitting requires good estimates of the errors in the measurements, and does not capture the effect of systematic deviations in data points. For example, visual inspection of the data suggests that the first data point of the 500 MHz data is displaced somewhat. Some of the bootstrap samples will not include this value, and some will have it replicated and its effect will therefore be manifested in the distribution of fits.

**Table 4:** R_2_ relaxation dispersion fitting parameters for the sample ^15^N data from BPTI

Residue	Field (MHz)	AIC	k_ex_	R_2_^c^	δ_ppmmin_	δ_ppm_^c^	p_A_
38	500	17.3	643 ± 11	8.18 ± 0.06	0.54 ± 0.01		
38^a^	500	17.2	643 ± 225	8.2 ± 1	0.54 ± 0.09		
38_Lit_	500		540 ± 10	8.26 ± 0.11	0.51 ± 0.01		
38	600	9.6	824 ± 18	8.28 ± 0.07	0.54 ± 0.01		
38_Lit_	600		766 ± 14	8.11 ± 0.11	0.53 ± 0.01		
38	500, 600	44.8	729 ± 10	8.31 ± 0.05^b^	0.54 ± 0.01		
38	500	31.8	195 ± 17	8.61 ± 0.04		1.56 ± 0.02	0.94 ± 0.003
38	600	8.7	461 ± 36	8.60 ± 0.06		1.42 ± 0.04	0.95 ± 0.001
38	500, 600	31.0	335 ± 12	8.61 ± 0.05^b^		1.45 ± 0.01	0.95 ± 0.001
38_Lit_	500, 600		380 ± 70	8.6 ± 0.1		0.91 ± 0.13	0.95 ± 0.004
38, 40	500, 600	61.7	322 ± 6	–		–	0.95 ± 0.001
12,16,17, 36,38,39, 40	500, 600	342.1	308 ± 3	–		–	0.95 ± 0.001

_Lit_Literature values (Millet et al. 2000)

^a^Errors calculated with non-parametric method

^b^Reported R_2_ value is the average of the R_2_ values at the two fields

^c^These parameters are residue specific so are not listed in this table when more than one residue is fit simultaneously

A significant contributor to the varied values of k_ex_ that are observed in the table is the correlation that is observed between parameters, as illustrated in Fig. 3. Performing simultaneous fits to data at two or more fields significantly reduces correlation between parameters, and results in error estimates that are more realistic. As can be seen in Table 4, fitting can be done for data at multiple fields and multiple residues. The two-field fit to the slow-exchange model gives a good fit to the data with little parameter correlation and relatively low error bars. Adding multiple residues further constrains the fit.

As a test of our ability to interactively simulate and fit data, we tested the software by trying to replicate fits done with NESSY (Bieri and Gooley 2011). R_2_ relaxation dispersion profiles were interactively simulated using the original parameters for the synthetic data models, one for fast exchange (Model 2) and one for slow exchange (Model 3) in the reference. To generate simulated data sets, the reference field was set to 800 MHz, the CPMG field strength values in the Options tab were set to the values used in the NESSY paper, and the error parameter was set to approximate one of the error levels used in the NESSY paper (5%). The sliders in the Simulate tab were then used to match the literature values for the parameters appropriate to each model. Simulated data sets were then generated by clicking the Gen button, and these were then fit using CMA-ES refinement and parametric bootstrapping with 500 samples. This protocol was repeated four times, and the parameters reported here are the average values from those replicates. Table 5 shows the results with the NESSY parameters used to generate the data, and the fitted parameters obtained both in NESSY and the software described here. As can be seen the fitting reproduces, within error limits, the parameters used to generate the data for both the Model 2 and Model 3 data. The error limits are similar to those obtained with NESSY. Though not shown here, AIC model selection chose the appropriate model (2 for the model 2 data, and 3 for the model 3 data). This example shows how the interactive simulation tools can be used to readily generate and fit sample data.

**Table 5:** R_2_ relaxation dispersion fitting parameters used in the interactive simulation

Model 2	k_ex_	R_2_	δ_ppmmin_	
Original^a^	3750	15.2	0.86	
Fit^b^	3815 ± 230	15.2 ± 0.3	0.84 ± 0.03	
NESSY fit^c^	3742 ± 188	15.5 ± 0.3	0.85	

Model 3	k_ex_	R_2_	δ_ppm_	p_A_
Original^a^	306.2	15.2	3.69	0.93
Fit^b^	317 ± 71	15.2 ± 0.2	3.70 ± 0.06	0.93 ± 0.02
NESSY fit^c^	330 ± 64	15.2 ± 0.1	3.78	0.93 ± 0.02

Data extracted from Fig. 2 of the NESSY paper (Bieri and Gooley 2011)

^a^Literature values used as input to generate simulated data

^b^Values from fit done with the software described here

^c^Values from NESSY as reported (Bieri and Gooley 2011)

#### Discussion

The software described here provides a unified system for analyzing multiple types of macromolecular dynamics with NMR spectroscopy. It combines, in a single package, tools for analyzing multiple types of relaxation experiments. Users do not have to choose from different programs for each type of experiment acquired. This is particularly important for those users that are not experts in the large number of NMR methods used for these investigations. At present the software can fit T_1_ and T_2_ exponential relaxation data, and R_2_ and R_1ρ_ relaxation dispersion, and CEST data. The software can analyze multiple types of data in one session and present the fits in a single window. The infrastructure is in place to expand this to other experiment types, such as DEST, and such implementation is in progress. The software does not yet support a number of the variants of the experimental types that can be analyzed with other software applications. For example, multiple quantum R_2_ relaxation dispersion, 3-site (or higher) exchange models, and accounting for pulse-sequence dependent artifacts are not yet supported. Still, for many users the software should provide a user-friendly way to analyze many data sets, and we are working towards a new release that will expand the range of models and experiment types that can be analyzed.

The program will become even more useful when the fits can be done simultaneously to different data sets where common parameters are constrained to be the same across the different experiment types. Having all the data and models in one program will facilitate this and is a major goal for a subsequent release.

An additional advance is the implementation of the tools in a user-friendly GUI-based program that runs on multiple platforms (Linux, Mac, Windows). Users are not forced to choose the software application based on their preferred OS. Installation is easy and does not require access to proprietary software such as MATLAB. While not necessary for use of the this software, we provide integration with NMRFx Analyst (the feature rich successor to NMRFx Processor (Norris et al. 2016)). Experimental data can be processed in NMRFx and appropriate data formats can be exported for analysis with the software described here. Together the two programs make for a rapid, integrated toolset for complete processing, visualization, and analysis of multiple types of NMR relaxation data.

The incorporation of simulation tools allows the software to be used in an educational setting where the influence of different relaxation parameters can be readily visualized. Availability of sample data allows users to test the software (and teach) with data that is known to work.
